# The effect of current antithrombotic therapy on mortality in nursing home residents with COVID-19: a multicentre retrospective cohort study

**DOI:** 10.1093/ageing/afae094

**Published:** 2024-05-15

**Authors:** Firdaouss Boutkourt, Thijs van Haaps, Reneé Brüggemann, Soerajja Bhoelan, Hugo ten Cate, Marieke J H A Kruip, Bart Spaetgens, Nick van Es, Tineke Roest, Karlijn J Joling, Karina Meijer, Jacqueline Hugtenburg

**Affiliations:** Department of Clinical Pharmacology and Pharmacy, Amsterdam UMC, location VUmc, De Boelelaan 1117, Amsterdam, The Netherlands; Farmadam Pharmacy Group, Contactweg 127, Amsterdam, The Netherlands; Department of Vascular Medicine, Amsterdam UMC, location AMC Meibergdreef 9, Amsterdam, The Netherlands; Pulmonary Hypertension & Thrombosis, Amsterdam Cardiovascular Sciences, Amsterdam, The Netherlands; Department of Internal Medicine, Maastricht University Medical Centre+, Maastricht, The Netherlands; Department of Hematology, UMC Groningen, University of Groningen, The Netherlands; Department of Internal Medicine, Maastricht University Medical Centre+, Maastricht, The Netherlands; Department of Hematology, Erasmus MC Erasmus University Medical Center Rotterdam, Rotterdam, The Netherlands; Department of Internal Medicine, Maastricht University Medical Centre+, Maastricht, The Netherlands; Department of Vascular Medicine, Amsterdam UMC, location AMC Meibergdreef 9, Amsterdam, The Netherlands; Pulmonary Hypertension & Thrombosis, Amsterdam Cardiovascular Sciences, Amsterdam, The Netherlands; Farmadam Pharmacy Group, Contactweg 127, Amsterdam, The Netherlands; Amsterdam Public Health Research Institute, Amsterdam UMC, VU University Medical Center, Amsterdam, The Netherlands, Department of Medicine for Older People, Amsterdam UMC, Location VUmc, De Boelelaan 1117, Amsterdam, The Netherlands; Department of Hematology, UMC Groningen, University of Groningen, The Netherlands; Department of Clinical Pharmacology and Pharmacy, Amsterdam UMC, location VUmc, De Boelelaan 1117, Amsterdam, The Netherlands

**Keywords:** nursing home residents, COVID-19, antithrombotic medication, mortality, anticoagulants, antiplatelets, older people

## Abstract

**Background:**

The first wave of COVID led to an alarmingly high mortality rate among nursing home residents (NHRs). In hospitalised patients, the use of anticoagulants may be associated with a favourable prognosis. However, it is unknown whether the use of antithrombotic medication also protected NHRs from COVID-19-related mortality.

**Objectives:**

To investigate the effect of current antithrombotic therapy in NHRs with COVID-19 on 30-day all-cause mortality during the first COVID-19 wave.

**Methods:**

We performed a retrospective cohort study linking electronic health records and pharmacy data in NHRs with COVID-19. A propensity score was used to match NHRs with current use of therapeutic dose anticoagulants to NHRs not using anticoagulant medication. The primary outcome was 30-day all-cause mortality, which was evaluated using a logistic regression model. In a secondary analysis, multivariable logistic regression was performed in the complete study group to compare NHRs with current use of therapeutic dose anticoagulants and those with current use of antiplatelet therapy to those without such medication.

**Results:**

We included 3521 NHRs with COVID-19 based on a positive RT-PCR for SARS-CoV-2 or with a well-defined clinical suspicion of COVID-19. In the matched propensity score analysis, NHRs with current use of therapeutic dose anticoagulants had a significantly lower all-cause mortality (OR = 0.73; 95% CI: 0.58–0.92) compared to NHRs who did not use therapeutic anticoagulants. In the secondary analysis, current use of therapeutic dose anticoagulants (OR: 0.62; 95% CI: 0.48–0.82) and current use of antiplatelet therapy (OR 0.80; 95% CI: 0.64–0.99) were both associated with decreased mortality.

**Conclusions:**

During the first COVID-19 wave, therapeutic anticoagulation and antiplatelet use were associated with a reduced risk of all-cause mortality in NHRs. Whether these potentially protective effects are maintained in vaccinated patients or patients with other COVID-19 variants, remains unknown.

## Key points

- Nursing home residents were severely affected during the first COVID-19 wave.
- We evaluated the potential effect of different types of antithrombotic therapy in nursing home residents (NHRs) with COVID-19.
- We performed a retrospective cohort study linking electronic health records and pharmacy data in NHRs with COVID-19.
- Therapeutic anticoagulation and antiplatelet use were associated with a reduced risk of 30-day all-cause mortality in NHRs.
- Our results underline the importance of continuing pre-existent use of antithrombotic therapy in NHRs with COVID-19.

## Introduction

The first waves of COVID-19 were associated with a high mortality rate and with a high incidence of thromboembolism [1–3], suggesting that (higher doses of) antithrombotic prophylaxis might be beneficial in hospitalised patients [1, 2]. During the pandemic, several randomized controlled trials evaluated intermittent or therapeutic-dose anticoagulation in hospitalised patients with COVID-19 [4–10]. Therapeutic anticoagulation was not beneficial among intensive care unit (ICU) patients [4–7], while clinical outcomes were improved in non-ICU patients when compared to standard of care [7, 9, 10]. In addition, multiple randomized controlled trials have shown that there is no proven efficacy of adding antiplatelet therapy (aspirin or P2Y12 inhibitors) to standard thromboprophylaxis or anticoagulant therapy in hospitalised patients with COVID-19 [11], although conceptually antiplatelet therapy, such as P2Y12 inhibitors, may still be an interesting therapeutic option in patients with COVID-19 [12].

Nursing home residents (NHRs) were severely affected during the COVID-19 pandemic. COVID-19 outbreaks in nursing homes led to mortality rates as high as 47% [13, 14]. Compared to older people residing in private homes, mortality rates among NHRs were 10–18-fold higher [15]. Several studies have shown that high age, impaired cognitive and physical function and co-morbidities, which are all highly prevalent in NHRs, are associated with increased mortality in COVID-19 [14, 16, 17]. Additionally, NHRs are at an increased risk of thrombotic complications [18], possibly making them also more susceptible to thrombotic complications associated with COVID-19 and thus further limiting their prognosis.

No trials with antithrombotic medication have been performed in NHRs with COVID-19. A few observational studies [13, 19] suggested a lower mortality among NHRs who were already receiving antithrombotic therapy for atrial fibrillation, venous thromboembolism, mechanical valves or other indications than those not using such medication, but the sample size was often small resulting in imprecise estimates and inability to stratify by type of antithrombotic treatment or adjust for confounding factors.

Therefore, the aim of the present study was to assess the effect of current antithrombotic therapy on 30-day all-cause mortality in a large Dutch cohort of NHRs with suspected or confirmed COVID-19 during the first COVID-19 wave.

## Material and methods

### Study design and setting

We performed a multicentre, retrospective cohort study in Dutch nursing homes using clinical data from the electronic health records (EHRs) (YSIS, GeriMedica). The data were collected as part of the national COVID-19 registration policy and were linked to the pharmacy data on medication use from the pharmacy information systems (Medimo). The Medical Ethics Committee of the AmsterdamUMC, location VUmc in Amsterdam reviewed, approved the study protocol and waived the need for informed consent for this retrospective study.

### Study population

The study population included NHRs from various nursing homes in the Netherlands. NHRs with clinically suspected COVID-19 were included from both somatic and psychogeriatric wards between 18 March 2020 and 13 May 2020. The diagnosis COVID-19 was preferably confirmed by real-time reverse-transcriptase polymerase chain reaction (RT-PCR) assay for SARS-CoV-2-mRNA in nasal or pharyngeal swabs. Due to limited testing capacity during the first COVID-19 wave in the Netherlands, not all NHRs could be tested [20]. In accordance with directives from the local health organisation (Gemeentelijke Gezondheidsdienst, GGD), NHRs clinically suspected of COVID-19 were therefore considered as positive if two other residents from the same location were already tested positive for COVID-19 [14, 21]. Testing of all NHRs clinically suspected of COVID-19 was implemented in the first week of April.

### Data collection

Age, sex, type of ward, co-morbidities and date of death were derived from the EHR. Data on co-morbidities consisted of dementia (including other cognitive disorders), chronic respiratory diseases, chronic cardiovascular disease, cerebrovascular disease, type 2 diabetes mellitus, chronic renal insufficiency and Parkinson’s disease. Data from the EHR were linked to the pharmacy data, which included data on antithrombotic use. Antithrombotic therapy was divided in three categories: therapeutic anticoagulants (low-molecular-weight heparin [LMWH] in therapeutic dose, vitamin K antagonists [VKAs] or direct oral anticoagulants [DOACs] in therapeutic dose), antiplatelet agents (e.g. aspirin, P2Y12 inhibitors or dipyridamole) and prophylactic-dose LMWH.

### Exposure

Current use of antithrombotic therapy was defined as the use during the last 30 days before COVID-19 diagnosis. In the primary analysis, exposed patients were defined as those receiving current anticoagulation (P1), i.e. LMWH in therapeutic dosage, VKAs or DOACs, who were compared to COVID-19 NHRs not receiving anticoagulant medication (P2). In the secondary analysis, the exposure was subdivided per each specific type of antithrombotic agent as follows: COVID-19 NHRs receiving anticoagulant medication with or without antiplatelet therapy (S1), those receiving antiplatelet therapy only (S2) and those not receiving any form of therapeutic anticoagulation and/or antiplatelet therapy (S3).

Patients in the control group who started anticoagulant medication during the follow-up period remained part of the control group for the entire follow-up period according to the intention-to-treat principle. Similarly, patients in the exposure group in whom anticoagulants were terminated during follow-up remained part of the exposure period for the entire follow-up period.

### Outcome

The main outcome was 30-day all-cause mortality. Follow-up started at either the date of confirmed positive RT-PCR for SARS-CoV-2 or the date of the first clinical suspicion for COVID-19 among patients not eligible for testing. Patients were censored at 30 days of follow-up.

### Statistical analysis

#### Primary analysis: propensity score model

For the primary analysis, a propensity score model was used to match NHRs on current anticoagulant medication 1:1 to patients not using anticoagulant medication using a nearest-neighbour matching approach. The propensity score was calculated using the following fourteen potentially confounding factors: age, sex, time in pandemic, type of ward (i.e. somatic vs. psychogeriatric unit vs. short stay/geriatric rehabilitation), acute heart failure, hypertension, diabetes mellitus, dementia, obesity, Parkinson’s disease, renal insufficiency, chronic lung disease, cardiovascular disease and cerebrovascular disease. Patient characteristics were compared between the matched exposed and non-exposed cohorts using standardised mean differences (SMDs). An SMD below 0.1 was considered to indicate a well-balanced and matched variable.

A logistic regression model was used to calculate odds ratios with 95% confidence intervals (CI) with the exposure as the only covariate. In addition, a marginal (unconditional) estimate was calculated using a regression model based on the complete cohort with patients weighted by their inverse probability of treatment using stabilised weights (IPW). For this IPW analysis, in order to overcome over-exposure, the trimming method was used [22], excluding patients with a propensity score below the 5th percentile and above the 95th percentile.

### Secondary analysis: logistic regression

Since in the secondary analysis three groups were compared, we did not use a propensity score matched analysis. Rather, 30-day mortality among the three groups was compared in univariate and multivariate logistic regression models. To assess the association between the current use of therapeutic dose anticoagulants (S1) and the use of antiplatelet agents (S2) of the NHRs with all-cause mortality, we performed a univariate logistic regression to show the naïve effect and a multivariate logistic regression including covariates (age, sex, dementia, cardiovascular disease, Parkinson disease and type of ward) in the model selection. Inclusion of co-variates in the multivariate logistic regression was based on a univariable association with a *P*-value below 0.10.

All analyses were performed in SPSS and R version 4.1.3 (R Foundation for Statistical Computing, Vienna, Austria; https://www.R-project.org).

## Results

### Study population and baseline characteristics

#### Study population before matching

**Figure 1:**
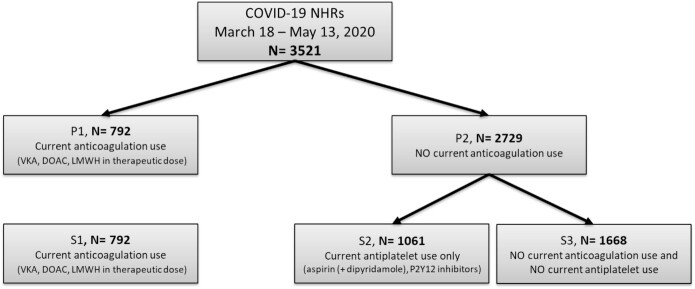
Study population before matching. Current use of antithrombotic therapy was defined as the use in the last 30 days before start of COVID-19.

During the study period due to limited testing capacity, 3521 NHRs tested positive for or had clinically suspected COVID-19 [20]. The baseline characteristics of the study population are shown in Table 1. 792 patients (22.5%) were current anticoagulant users (groups P1 and S1), and 2729 (77.5%) were non-anticoagulant users (group P2). Of these 2729 non-anticoagulant users, 1061 (30.1% of the total population) used antiplatelet agents (group S2) (Figure 1).

**Table 1:** Baseline characteristics of the total study population before propensity score matching (1:1)

	NHRs with current^a^ use of therapeutic dose anticoagulant (P1, before 1:1 propensity score matching) (*n* = 792)	NHRs with no current use of therapeutic dose anticoagulant (P2, before 1:1 propensity score matching) (*n* = 2,729)	*P-*value	SMD
Demographical data				
Age (mean in years), med (IQR)	86 [82, 91]	83 [78, 90]	<0.001	0.298
Sex, *n* (%) Male Female	326 (41.2) 466 (58.8)	978 (35.9) 1751 (64.2)	0.008	0.108
Clinical data Co-morbidities (*n*, %)				
Congestive heart failure	13 (1.6)	8 (0.3)	<0.001	0.138
Hypertension	0 (0.0)	1 (0.0)	1.000	0.027
Obesity	26 (3.6)	43 (1.8)	0.005	0.112
Dementia	417 (53.0)	1563 (58.1)	0.013	0.102
Cardiovascular disease	646 (82.1)	954 (35.4)	<0.001	1.075
Cerebrovascular disease	402 (51.1)	901 (33.5)	<0.001	0.361
Diabetes mellitus	206 (26.2)	626 (23.3)	0.099	0.068
Pulmonary disease	171 (21.7)	443 (16.5)	0.001	0.134
Renal insufficiency	180 (22.9)	423 (15.7)	<0.001	0.184
Parkinson’s disease	45 (5.7)	179 (6.6)	0.389	0.039
Type of ward (*n*, %)			<0.001	0.302
Psychogeriatrics	311 (39.3)	1337 (49.0)		
Somatics	219 (27.7)	447 (16.4)		
Short-term stay^b^	134 (16.9)	406 (14.9)		
Other ward, not specified	128 (16.1)	539 (19.8)		
Time in pandemic, days (mean)	44.69 (13.72)	44.31 (13.75)	0.492	0.028
Days to event (mean, SD)	25.73 (15.87)	24.32 (16.02)	0.029	0.302
Outcome (*n*, %)				
All- cause mortality^c^	179 (22.6)	708 (26.0)	0.061	0.079

NHRs, nursing home residents.

^a^Current use was defined as the use in the last 30 days before start of COVID-19.

^b^Primary care and geriatric rehabilitation.

^c^Mortality within 30 days after COVID-19 diagnosis.

### Study population for the propensity score

**Figure 2:**
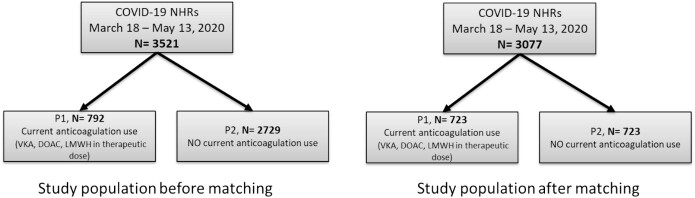
Study population before and after matching. Current use of antithrombotic therapy was defined as the use in the last 30 days before start of COVID-19.

In total, the propensity score using the described covariates above could be calculated for 3077 patients (87.4%) of whom 723 (23.5%) used therapeutic anticoagulation. Based on the propensity score, these 723 NHRs using therapeutic anticoagulants (P1) could be matched to 723 NHRs not using therapeutic anticoagulation (P2) prior to COVID-19 (Figure 2).

### Baseline characteristics before and after matching

Before matching, the NHRs using current therapeutic dose anticoagulants (group P1) were on average older (86 vs. 83 years; *P* < 0.001) than those without current use of therapeutic dose anticoagulants (group P2). The current anticoagulant using NHRs had less co-morbidities compared to group P2 (Table 1). All-cause mortality was 22.6% (*n*/*N* = 179/792) in group P1 and 26.0% (*n*/*N* = 708/2729) in group P2.

In the matched population, the all-cause mortality in group P1 was 24.2% (*n*/*N* = 175/723) and 30.4% (*n*/*N* = 220/723) in group P2 (Table 2).

**Table 2:** Baseline characteristics of the total study population after propensity score matching (1:1)

	NHRs with current^a^ use of therapeutic dose anticoagulant (P1, after 1:1 propensity score matching) (*n* = 723)	NHRs with no current use of therapeutic dose anticoagulant (P2, after 1:1 propensity score matching) (*n* = 723)	*P-*value	SMD
Demographical data				
Age (mean in years), med (IQR)	85.7 (7.4)	85.4 (8.08)	0.394	0.045
Sex, *n* (%) Male Female	293 (40.5) 430 (59.5)	275 (38.0) 448 (62.0)	0.360	0.051
Clinical data Co-morbidities (*n*, %)				
Congestive heart failure	12 (1.7)	1 (0.1)	0.005	0.162
Hypertension	0 (0)	0 (0)	NA	<0.001
Obesity	26 (3.6)	21 (2.9)	0.553	0.039
Dementia	416 (57.5)	426 (58.9)	0.631	0.028
Cardiovascular disease	645 (89.2)	645 (89.2)	1.000	<0.001
Cerebrovascular disease	400 (55.3)	407 (56.3)	0.751	0.019
Diabetes mellitus	205 (28.4)	214 (29.6)	0.643	0.027
Pulmonary disease	171 (23.7)	161 (22.3)	0.574	0.033
Renal insufficiency	180 (24.9)	163 (22.5)	0.323	0.055
Parkinson’s disease	44 (6.1)	51 (7.1)	0.524	0.039
Type of ward (*n*, %)			0.038	0.168
Psychogeriatrics	286 (39.6)	324 (44.8)		
Somatics	204 (28.2)	153 (21.2)		
Short-term stay^b^	120 (16.6)	123 (17.0)		
Other ward, not specified	7 (1.0)	7 (1.0)		
Time in pandemic (mean)	42.6 (12.4)	42.6 (11.89)	0.918	0.005
Days to event (mean, SD)	27.19 (15.55)	25.05 (15.88)	0.010	0.137
Outcome (*n*, %)				
All- cause mortality^c^	175 (24.2)	220 (30.4)	0.009	0.140

NHRs, nursing home residents.

^a^Current use was defined as the use in the last 30 days before start of COVID-19.

^b^Primary care and geriatric rehabilitation.

^c^Mortality within 30 days after diagnose COVID-19.

### Primary analysis

For the primary analysis, group P1 (current use of therapeutic dose anticoagulants) was compared to group P2 (no current use of therapeutic dose anticoagulants). Prior to matching, NHRs in group P1 showed a lower all-cause mortality (OR = 0.83; 95% CI 0.69–1.00) as compared to group P2 during a follow-up of 30 days.

**Figure 3:**
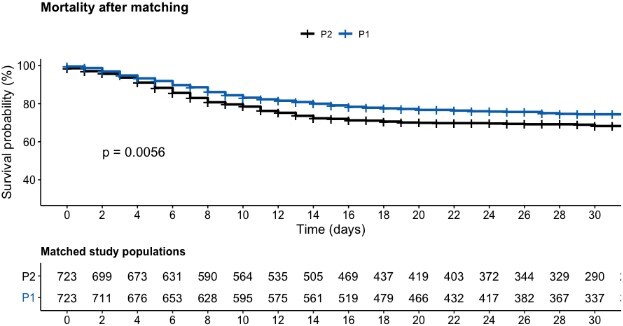
Survival probability curve after matching.

Matched group P1 had a significantly lower all-cause mortality (OR = 0.73; 95% CI 0.58–0.92) as compared to matched group P2 during a follow-up of 30 days (Figure 3).

Next to this, an inverse probability weighting using the calculated propensity score was also analysed. In NHRs using therapeutic anticoagulants, there was a significant association with a lower all-cause mortality (OR 0.70; 95% CI 0.63–0.79) as compared to NHR non-users (2,354) during a follow-up of 30 days. There was no significant association found between all-cause mortality and the use of antiplatelet therapy compared to patients not using antiplatelet therapy (OR = 0.87; 95% CI 0.76–1.01).

### Secondary analysis

For the secondary analysis, groups S1, S2 and S3 were compared. The baseline characteristics between these three groups are presented in Table 3. The NHRs with current use of therapeutic dose anticoagulants (group S1) were on average older than those with current use of antiplatelet agents (group S2) and the control group (group S3). The NHRs in group S1 and S2 had more co-morbidities compared to NHRs in group S3, including cardiovascular disease, cerebrovascular disease, diabetes mellitus, pulmonary disease and renal insufficiency. All-cause mortality was 26.3% (*n* = 438) in group S3 and 22.6% (*n* = 179) and 25.4% (*n* = 270) in group S1 and group S2, respectively.

**Table 3:** Baseline characteristics of the total study population for the secondary analysis

	NHRs with current^a^ use of therapeutic dose anticoagulant (S1) (*n* = 792)	NHRs with current use of antiplatelet therapy only (S2) (*n* = 1,061)	NHRs with no current use of therapeutic anticoagulant + no current use of antiplatelet therapy (S3) (*n* = 1,668)	*P-*value
Demographical data				
Age (years), med (IQR)	86 [82, 91]	85 [80, 90]	84 [77, 90]	
Sex, *n* (%) Male Female	326 (41.2) 466 (58.8)	422 (39.8) 639 (60.2)	557 (33.4) 1109 (66.6)	<0.001
Clinical data Co-morbidities (*n*, %)				
Dementia	417 (53.0)	600 (56.8)	963 (58.9)	0.022
Cardiovascular disease	646 (82.1)	613 (58.0)	341 (20.9)	<0.001
Cerebrovascular disease	402 (51.1)	644 (60.9)	257 (15.7)	<0.001
Diabetes mellitus	206 (26.2)	307 (29.0)	319 (19.5)	<0.001
Pulmonary disease	171 (21.7)	213 (20.2)	230 (14.1)	<0.001
Renal insufficiency	180 (22.9)	213 (20.2)	210 (12.8)	<0.001
Parkinson’s disease	45 (5.7)	67 (6.3)	112 (6.9)	0.561
Type of ward (*n*, %)				<0.001
Psychogeriatrics	311 (39.3)	505 (47.6)	834 (50.1)	
Somatics	219 (27.7)	253 (23.8)	194 (11.6)	
Short-term stay^b^	134 (16.9)	134 (12.6)	272 (16.3)	
Other ward, not specified	128 (16.1)	169 (16.0)	366 (22.0)	
Outcome (*n*, %)				
All-cause mortality^c^	179 (22.6)	270 (25.4)	438 (26.3)	0.135

Subdivided into three groups: current use of therapeutic dose anticoagulant (S1), current use of antiplatelet therapy only (S2), and no current use of therapeutic dose anticoagulant + no current use of antiplatelet therapy (=S3)

NHRs, nursing home residents.

^a^Current use was defined as the use in the last 30 days before start of COVID-19.

^b^Primary care and geriatric rehabilitation.

^c^Mortality within 30 days after COVID-19 diagnosis.

The univariate logistic regression analysis showed that the all-cause mortality at 30 days after diagnosis COVID-19 in group S1 was 18% lower compared to group S3 (OR = 0.82, 95% CI = 0.67–1.00; *P* = 0.046) and that the all-cause mortality at 30 days after diagnosis COVID-19 in group S2 was 4.5% lower compared to group S3 (OR = 0.96, *P* = 0.605, 95% CI = 0.80–1.14). The multivariate logistic regression showed that the all-cause mortality at 30 days after diagnosis COVID-19 in group S1 was 38% lower compared to group S3 (OR = 0.62, 95% CI = 0.48–0.82; *P* < 0.001) and that the all-cause mortality at 30 days after diagnosis COVID-19 in group S2 was 20% lower compared to group S3 (OR = 0.80, *P* = 0.044, 95% CI = 0.64–0.99).

## Discussion

In this retrospective cohort study, we assessed the effect of the current use of therapeutic dose anticoagulants or antiplatelet agents on the 30 day all-cause mortality in NHRs with COVID-19 during the first wave of the pandemic. Although we did not conduct a formal comparison between individuals with positive COVID PCR and those without, previous research in the NH population during the first wave, using the same database, indicated that although there was symptom overlap (which argued for the use of a PCR), there were also a number of differences (including with regards to fever) [13, 14, 21]. After propensity score matching, NHRs using therapeutic anticoagulants had a significantly lower all-cause mortality (OR = 0.73; 95% CI 0.58–0.92) compared to NHRs who did not use therapeutic dose anticoagulants. The secondary analysis showed that both therapeutic anticoagulant use and antiplatelet therapy were associated with decreased mortality, with odds ratios of 0.62 (CI: 0.48–0.82) and 0.80 (0.64–0.99), respectively.

This study addresses the knowledge gap on the potential effect of different types of antithrombotic therapy on mortality in NHRs with COVID-19. Few other, smaller studies evaluated the effect of current use of antithrombotic therapy on mortality in NHRs and older people with COVID-19. A US study also found lower odds of 30-day mortality in 1,253 NHRs with COVID-19 who received current antithrombotic therapy (defined as antiplatelet therapy as well as therapeutic anticoagulants such as warfarin or DOACs) [19]. The limited sample size precluded a stratified analysis of the different medication classes. Another Dutch nursing home study evaluated the effect of oral antithrombotic therapy in NHRs with COVID-19 and found no significant difference in all-cause mortality [13]. This was, however, a small study (*n* = 101) in which univariate analyses did show a slight but non-significant benefit for those using oral antithrombotic therapy.

Two larger, population-based, studies in community-dwelling older people found a protective effect on mortality in older people with pre-existent antithrombotic therapy, similar to our study [23, 24]. All-cause mortality was significantly higher among non-anticoagulated patients as compared to patients treated with therapeutic antithrombotic therapy [24], and chronic DOAC intake was also found to be associated with a decreased mortality risk [23]. Although most studies suggest a favourable effect of antithrombotic therapy, surprisingly, opposite results have also been published. In a study assessing the effect of VKA on mortality in older patients hospitalised to a geriatric acute care unit [25], those using VKA seemed to have shorter survival times than others [26].

This study has strengths and limitations. Our study is an important study for ‘pandemic preparedness’, well-organised and co-ordinated observational studies, collecting relevant parameters, may help to guide treatment and guideline choices. A strength of this study was the ability to assess the effect of different medication classes on mortality in the frail COVID-19-positive nursing home population. We were able to include a large sample by making use of linkage of electronic patient files from different databases. This provided us with valuable data including the frailest NHRs that are not represented in most trials. This large data set allowed us to perform extensive analyses and to correct for a multitude of confounding factors. Nevertheless, we cannot exclude the possibility of residual confounding. We were limited in the number of available confounding factors in the collected data. However, the use of propensity score matching provided us with the ability to adjust for unmeasured confounding. It should be also recognised that NHRs with limited life expectancy are more likely to stop antithrombotic medication. However, our baseline characteristic gives no indication that this is the case. The data indeed show that, similar to other populations, more co-morbidity is associated with more antithrombotic drugs.

An important limitation is that all data were collected during the first COVID wave limiting its generalizability to later phases of the COVID pandemic. Moreover, not all NHRs could be tested, due to limited testing capacity during the first COVID-19 wave in the Netherlands. Mortality during this first wave was extremely high in the nursing home population and is thought to have been higher than in any of the subsequent waves. Most likely, the explanation for this is multifactorial. Improvements in treatment for COVID-19 are unlikely to have changed the course of the disease in NHRs, as most will not be referred for hospital care. Additionally, mutation of the SARS-CoV-2 virus resulted in different virus variants with significantly lower impact on mortality and possibly also with less thrombogenic potential. The start of the vaccination programme on 6 January 2021 in the Netherlands will most likely also have had an effect on mortality and the risk for thromboembolic complications, although it is almost impossible to correctly quantify this effect. It is unclear whether or not our results would be reproducible in the current situation. We plan on expanding our analyses to subsequent waves in future analyses. Additionally, follow-up did not start at the same time for PCR-confirmed NHRs and COVID-19-suspected NHRs, which, in theory, could have resulted in immortal time bias. However, the time span is very limited and sensitivity analyses with only PCR-confirmed NHRs showed similar results (data not shown).

## Conclusion

This study shows that current use of antithrombotic therapy in NHRs with COVID-19 was associated with a lower 30-day mortality risk. This suggests that antithrombotic agents, possibly by virtue of limiting thrombo-inflammatory organ damage, contribute to reducing death rate in this specific, frail population. With our study being the only one in this frail population and because this has been an association study, we must exert caution in proposing a change in the current treatment guidelines for the prevention of VTE in NHRs with COVID-19. Our results underline the importance of continuing pre-existent use of antithrombotic therapy in NHRs with COVID-19. Future research should mainly focus on larger-scale prospective, randomized studies in NHRs in order to formulate recommendations with regards to the best treatment regime to prevent VTE in NHRs with COVID-19 or other systemic infectious diseases.

## Supplementary Material

aa-23-0859-File007_afae094
