# Supplementary material for: The effect of current antithrombotic therapy on mortality in nursing home residents with COVID-19: a multicentre retrospective cohort study
Source: Age Ageing. 2024 May 15;53(5):afae094. doi: 10.1093/ageing/afae094 (PMC11095411; doi:10.1093/ageing/afae094)
Supplement: aa-23-0859-File007_afae094 [file aa-23-0859-file007_afae094.docx]

**The effect of current antithrombotic therapy on mortality in nursing home residents with COVID-19: a multicentre retrospective cohort study**


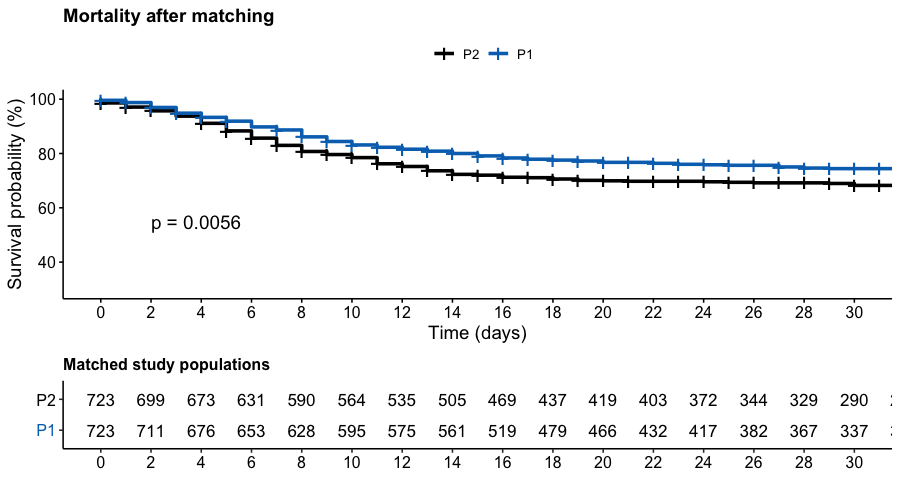


**Figure 3**. Survival probability curve after matching
